# Integrating beneficial microorganisms and soil amendment for grapevine health: toward eco-friendly seasonal fungal disease management and soil improvement

**DOI:** 10.3389/ffunb.2025.1713132

**Published:** 2025-12-10

**Authors:** Lobna Hajji-Hedfi, Takwa Wannassi, Samar Dali, Wissem Hamdi, Boulbaba L’taief, Ahmed M. Abdel-Azeem

**Affiliations:** 1 Regional Centre of Agricultural Research of Sidi Bouzid, Sidi Bouzid, Tunisia; 2 Laboratory of Agriculture Production Systems and Sustainable Development (LR03AGR02), Department of Agricultural Production, Higher School of Agriculture of Mograne, University of Carthage, Mograne-Zaghouan, Tunisia; 3 Higher Institute of the Sciences and Techniques of Waters of Gabes, Gabes University, Gabes, Tunisia; 4 Biology Department, College of Sciences in Abha, King Khalid University, Abha, Saudi Arabia; 5 Department of Botany and Microbiology, Faculty of Science, Suez Canal University, Ismailia City, Egypt; 6 Research Institute of the University of Bucharest (ICUB), Bucharest, Romania; 7 Centre for Mineral Biogeochemistry, Faculty of Natural and Agricultural Sciences, University of the Free State, Bloemfontein, South Africa

**Keywords:** bio-stimulant, compost, grapevine, *Pseudomonas yamanorum*, *Trichoderma longibrachiatum*

## Abstract

This study evaluates the effectiveness of two biological agents, *Trichoderma longibrachiatum* and *Pseudomonas yamanorum*, together with compost addition. The results show that combining compost with the microbial consortium enhances the physicochemical properties of the soil by increasing nitrogen, carbon, and organic matter while reducing bulk density and nitrate levels. Furthermore, this combination stimulates plant defense mechanisms, leading to increased antioxidant enzyme activity and higher phenolic compound levels. The amendments also improved critical soil properties, increasing organic matter (up to 4.14%), organic carbon (up to 2.40%), and total nitrogen (up to 1.47 mg/g), suggesting that these effects may be linked to the richness of microorganisms in the compost. The combined treatments also reduced the symptoms of fungal diseases: the severity of *Botrytis cinerea* decreased from 82%–92% to 4.97%–7.71%; *Erysiphe necator* from 89%–95% to 2.34%–8.03%; and *Plasmopara viticola* from 70%–95% to 2.84%–5.66%. In conclusion, the use of compost and beneficial microorganisms as bio-stimulants could offer an effective and sustainable solution for improving grapevine soil quality and managing fungal diseases.

## 1 Introduction

*Vitis vinifera* L. is a climbing woody plant with a perennial stem (Pallas, 2009; Parker et al., 2013). It is a dicotyledonous angiosperm belonging to the *Vitaceae* family (Planchon, 1887). The vine is considered one of the most cultivated fruit plants in the world. In addition to fresh fruit, it is used in the production of multiple products such as wine, vinegar, dried fruits, and juices (Meng et al., 1999). However, like most species of agronomic interest, *Vitis Vinifera* vines cultivated since antiquity have been selected over time to maintain yield and quality according to local needs. To meet growing industrial and consumer demands, viticulture resorted to the massive use of phytosanitary products and fertilizers, a practice that continues today (Visconti et al., 2024). In recent years, studies have demonstrated that the excessive use of chemical fungicides has harmful effects. These chemicals contribute to environmental pollution and pose several health risks to humans and animals. In addition, their intensive use is costly and threatens the long-term sustainability of cultivation systems (Okagu et al., 2023). Biological consequences have also been observed, as repeated application promotes the emergence of new resistant pathogen strains and affects the biology of pest populations (Gisi and Sierotzki, 2008; Chen et al., 2007; Gisi et al., 2007; Baudoin et al., 2008; Furuya et al., 2010). Moreover, throughout the grapevine’s vegetative cycle, it is subjected to numerous fungal diseases that affect both production quality and quantity.

Among the most threatening diseases in this crop are those caused by *Botrytis cinerea* (gray mold), *Erysiphe necator* (powdery mildew), and *Plasmopara viticola* (downy mildew) (Hajji-Hedfi et al., 2025b). Downy mildew is caused by an obligate biotrophic parasite that requires a living host to develop, grow, and survive, attacking all herbaceous tissues of the vine, including branches, leaves, clusters, and tendrils (Yin et al., 2017). When climatic conditions favor the development of *P. viticola*, the resulting damage is economically significant. Yield can be considerably reduced following infection (Gai and Wang, 2024). Similarly, powdery mildew is a major disease affecting white, red, and blue cultivars. It is caused by the obligate biotrophic pathogen *E. necator*, which is strictly associated with *Vitaceae* genera such as *Ampelopsis, Cissus, Parthenocissus*, and *Vitis* (Qiu et al., 2015). This disease substantially reduces fruit quality and yield by infecting all host tissues. Early infections in extreme circumstances, destroyed the grape fruits leading to a change in the chemical compounds of grapes and result in unfavorable flavors in the finished product, even minor infections have been demonstrated to have a detrimental effect on wine quality (Rousseau et al., 2008). The ascomycete *B. cinerea*, a necrotrophic fungus that enters plant tissues through wounds or natural openings, causes gray mold by killing host cells and colonizing the affected area. It is particularly harmful to ripening berries and can lead to major production losses and a notable decline in wine quality. Infection often begins during flowering and remains latent until fruit maturity (Dubos, 2002; Mouria et al., 2013).

The disease can cause yield losses of up to 40%, depending on environmental conditions and vineyard management. Even low infection levels (1%–3% of grapes) can affect wine quality due to the production of undesirable compounds and contaminated grapes may exhibit reduced sugar, acid, and polyphenol levels, resulting in wines with diminished color, aroma, and taste (Jiang et al., 2023).

Intensive fungicide use in vineyards has led to significant phytosanitary challenges and environmental concerns. The excessive application of fungicides to control diseases such as downy mildew and gray mold has resulted in soil contamination, degradation of soil microflora, and the emergence of fungicide-resistant pathogen strains. Sustainable approaches that prioritize environmental and human health have thus become necessary. Biological control, using organic or microbial sources, has emerged as a promising alternative for vineyard disease management. Studies have demonstrated that microbial biological control agents (BCAs), such as beneficial bacteria and fungi, can effectively suppress pathogens and reduce reliance on chemical fungicides (Hajji-Hedfi et al., 2023a; Hajji-Hedfi et al., 2023b; Hajji-Hedfi et al., 2025b; Hajji-Hedfi et al., 2025a).

The International Organization for Biological Control defines biological protection as “the use of living organisms in order to prevent or reduce the damage caused by pests” (Lepoivre, 2003). This approach involves the use of natural enemies such as predators and parasitoids to suppress harmful organisms, including plant pathogens (Sutton, 1995). Several biocontrol products include antagonistic microorganisms, plant extracts, and natural substances. For instance, natural molecules such as chitosan or cysteine can inhibit mycelial growth of several fungi associated with vine diseases, both *in vitro* and in planta (Octave, 2005; Nascimento et al., 2007).

Biological protection of grapevines against fungal pathogens can be achieved using microbial agents originating from the rhizosphere, such as *Trichoderma* species (Fourie and Halleen, 2005; Amreen and Kumar, 2012; Lee et al., 2012; Tuao Gava and Leal Menezes, 2012; Silva-Valderrama et al., 2021). These fungi are widely recognized as effective biofungicides due to their antagonistic activity against various pathogens (Di Marco et al., 2004; Kotze et al., 2011; Pollard-Flamand et al., 2022). *Trichoderma* species exert biocontrol through competition for nutrients and space, production of antimicrobial compounds, and induction of plant defense responses. Their application in viticulture not only assists disease management but also aligns with sustainable agricultural practices by reducing dependence on chemical fungicides. Although not all mechanisms are fully understood, they are generally associated with mycoparasitism, production of inhibitory compounds, spatial and nutrient competition with pathogenic fungi, stimulation of plant growth, and enhancement of host resistance (Hajji-Hedfi et al., 2025b). Alongside beneficial fungi, several bacteria have been used as biocontrol agents against vine diseases (Ferreira et al., 1991; Schmidt et al., 2001; Compant et al., 2013; Hajji-Hedfi et al., 2025c). These bacteria, along with their secondary metabolites, can inhibit or reduce the growth of pathogens associated with vine wood diseases. Their modes of action may include antibiosis, competition for niches and nutrients, and stimulation of the host plant’s immune system (Lugtenberg and Kamilova, 2009; Compant et al., 2013; Hajji-Hedfi et al., 2025a).

This study aimed to assess the antifungal potential of the BCAs *T. longibrachiatum, P. yamanorum*, and compost amendment either separately or in combination across various phenological stages (dormancy, vegetative stage, fruit set) in a grapevine field experiment involving economically important cultivars (Victoria, Superior Seedless, and Early Sweet) in Tunisia. Additionally, the study aimed to evaluate seasonal impacts of the treatments. Disease severity caused by *B. cinerea, E. necator*, and *P. viticola* was assessed. Numerous parameters were measured, including biochemical defense responses (phenolic contents), oxidative stress markers (e.g., malondialdehyde (MDA) levels), antioxidant enzyme activities (APX, POX, and CAT), and physiological factors (chlorophyll content). Based on their potential biocontrol activity in the field and their successful isolation at the Plant Protection and Biological Sciences Laboratory at the Regional Center for Agricultural Research (CRRA) in Tunisia, *T. longibrachiatum* and *P. yamanorum* were selected (Hajji-Hedfi et al., 2025b). This comprehensive evaluation aims to support a biologically sound, long-term strategy for improving grapevine health, yield, and agroecosystem resilience, which may be integrated into future regenerative and sustainable practices.

## 2 Materials and methods

### 2.1 Experiment design

**Figure 1:**
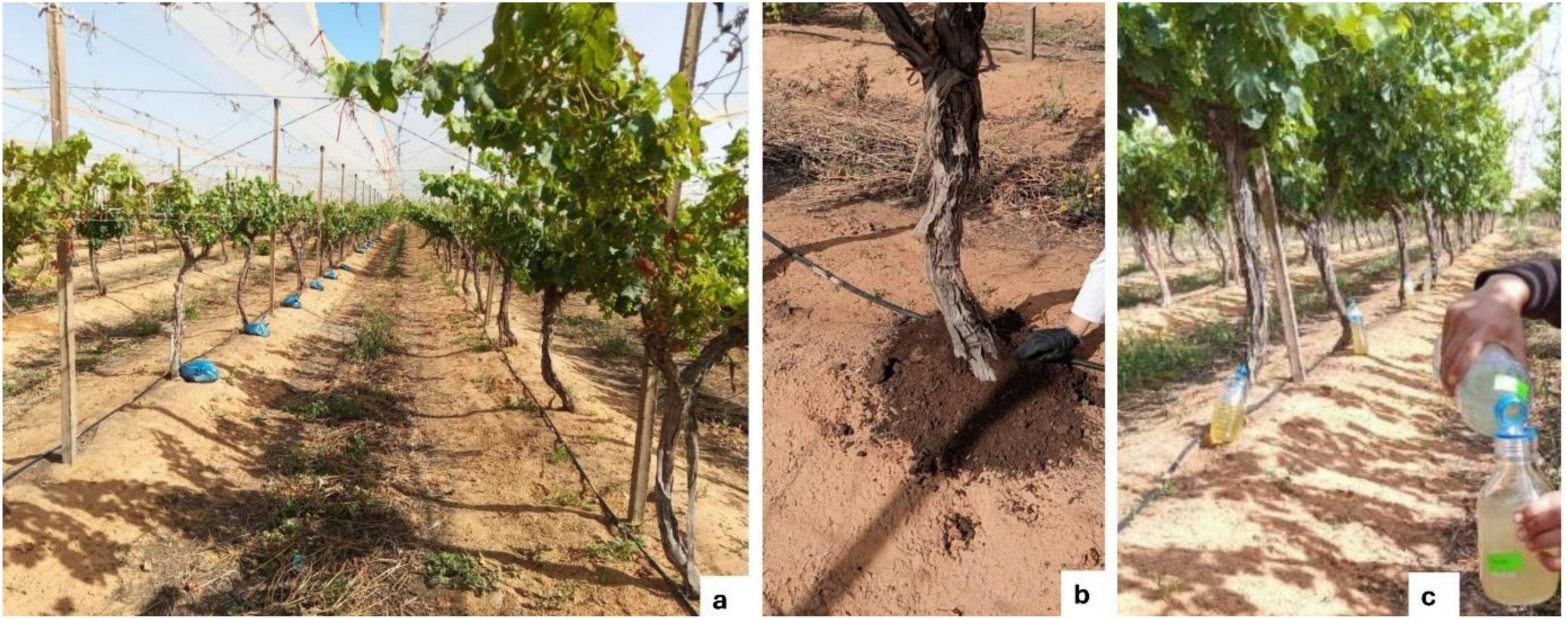
Field application of treatments to the grapevines: **(a)** grapevines experimental plots; **(b)** compost application; and **(c)** in-field preparation of microbial consortium to apply in vines.

The study was conducted in a 6-year-old experimental vineyard located in the Regueb region in the Sidi Bouzid governorate, Tunisia (35.03824° latitude, 9.484263° longitude), in 2023. Three *Vitis vinifera* L. cultivars Victoria, Early Sweet, and Superior Seedless were cultivated in this area. Treatments in the vineyard followed a completely randomized block design (CRBD) with four replicate plots per treatment, each consisting of six plants (**Figure 1**). The treatments were: T1, compost only; T2, compost + microbial consortium; T3, microbial consortium only; and T4, negative control. Treatments were applied once during each phenological stage: dormancy (P1), vegetative stage (P2), and fruit set (P3). Sixty days after the final application, the vineyard was monitored, and soil and leaf samples were collected to evaluate treatment effects.

The two BCAs used in this study consisted of a microbial consortium of *Trichoderma longibrachiatum* (GenBank accession LT707585) and *Pseudomonas yamanorum* (GenBank accession PQ555427). Both BCAs were obtained from the Plant Protection and Biological Sciences Laboratory at the CRRA in Sidi Bouzid, Tunisia, at concentrations of 10^6^ spores/ml for the beneficial fungus and 10^8^ CFU/mL for the rhizobacteria (Hajji-Hedfi et al., 2023b). The compost was produced at the CRRA using vineyard pruning residues and organic waste, as described by (Hajji-Hedfi et al., 2025b). Microbial suspension treatments were prepared according to the method described by (Hajji-Hedfi et al., 2025b). A dose of 0.5 L of each microbial suspension and 4 kg of compost per plant was applied per grapevine plant. 

Field screening of fungal and fungal-like pathogens was conducted to assess the sanitary status of the experimental site. The identified diseases *B. cinerea, E. necator*, and *P. viticola* were confirmed based on descriptions reported by (Hajji-Hedfi et al., 2025b).

#### 2.1.1 Sampling

Leaf and soil samples were collected at three phenological stages dormancy (P1), vegetative stage (P2), and fruit set (P3) 60 days after treatment application. For each plot per treatment, representative soil samples were taken from six vines within the plot and composited into a single representative sample (n = 4 samples per treatment). Soil samples were collected from the topsoil layer using a sterile shovel, placed in labeled plastic zip-lock bags, and transported to the laboratory. Samples were air-dried at 37°C, sieved, and homogenized for laboratory analysis. Leaf samples were randomly selected from fully expanded leaves at similar developmental stages from different vines (n = 24 per treatment per plot), placed in sterile bags, and transported to the laboratory, where they were stored at 4°C until analysis.

### 2.2 Soil physicochemical analysis

Several soil parameters were assessed to evaluate the effects of the microbial consortium and compost on soil quality, including bulk density, pH, nitrate content, organic carbon and organic matter content, and total nitrogen. Bulk density was measured by inserting a 100 cm³ cylinder into the soil, air-drying the sample at room temperature for 6 days, and weighing it to calculate mass per unit volume. Soil pH was measured according to the AFNOR X 31–103 standard using a calibrated pH meter (Hanna Instruments, Virginia, USA). Nitrate content was determined in mg/kg using a nitrate meter (LAQUA-twin, Germany). Organic carbon and organic matter were assessed following the method described by (Walkley and Black, 1934), and total nitrogen was determined using the method employed by (Kjeldahl, 1883).

### 2.3 In-field evaluation of microbial consortium and compost treatments efficacy against fungal diseases

In the field, symptoms of *B. cinerea*, *E. necator*, and *P. viticola* were assessed on all six plants within each plot (n = 24) using a scale from 0 to 4. A score of 0 indicates healthy leaves; 1 indicates that 1%–25% of grapevine leaves are affected; 2 indicates that 26%–50% of leaves are infected; 3 corresponds to 51%–75% infected leaves; and 4 indicates that more than 75% of leaves are affected (Brito et al., 2021). The Disease Severity Index (DSI) was calculated using McKinney’s formula (Equation 1), as follows:

(1) $$\text{DSI }(\%)=\frac{\text{Σ }(\text{v}\times \text{n})\;}{\left(\text{N}\times \text{V}\right)}\times \;100\;$$

where v is the numerical value assigned to the disease class, n is the number of grapevine plants in each class, N is the total number of plants, and V is the highest value on the disease scale (Hajji-Hedfi et al., 2025b).

### 2.4 Assessment of physiological and biochemical parameters

#### 2.4.1 Chlorophyll content

Chlorophyll content in grapevine leaves was measured in SPAD units using a SPAD-502Plus portable chlorophyll meter (Konica Minolta, Japan). Measurements were taken on four fully expanded leaves per plant (n = 24) for each cultivar and treatment at each sampling time to evaluate treatment effects on photosynthetic activity across the different phenological stages.

#### 2.4.2 Enzymatic activities and stress-related parameters

To evaluate the effects of treatments on stress-related parameters, enzymatic activities and biochemical markers were measured in grapevine leaves. Parameters evaluated included catalase (CAT), peroxidase (POX), ascorbate peroxidase (APX), total phenolic content, malondialdehyde (MDA) content, and total protein content to understand biochemical changes.

For enzyme extraction, leaf samples were homogenized in an extraction buffer containing 0.1 M phosphate buffer (pH 7), 1 mM ethylenediaminetetraacetic acid (EDTA) (Sigma-Aldrich, St. Louis, USA), and 1% polyvinylpyrrolidone (PVP) (Sigma-Aldrich, St. Louis, USA). The homogenate was centrifuged at 12,000 × g for 20 min at 4°C. The supernatant was then collected and stored at –20°C for subsequent analyses. CAT activity was measured following the method of (Aebi, 1984). POX activity was assessed by measuring absorbance at 420 nm according to (Velazhahan and Vidhyasekaran, 1994), using a solution containing 1.5 ml pyrogallol (Merck, Darmstadt, Germany), 0.5 ml enzymatic extract, and 0.5 ml H_2_O_2_. APX activity was measured by absorbance at 290 nm following (Singleton and Rossi, 1965), using a solution composed of 890 μl buffer, 2 μl ascorbate (Merck, Darmstadt, Germany), 30 μl enzymatic extract, and 20 μl H_2_O_2_. Total protein content was determined using the method described by (Bradford, 1976). Protein solution was prepared by mixing 5 μl protein extract, 200 μl Bradford reagent (Bio-Rad, Hercules, CA), and 795 μl phosphate buffer, and absorbance was measured at 595 nm. Total phenolic content was measured using the Folin–Ciocalteu method as described by (Bradford, 1976). MDA content was determined using the method of (Haraguchi et al., 1995).

### 2.5 Statistical analysis

Data were analyzed using R software (RStudio Version 2024.12.0 + 467). Data normality was assessed using the Shapiro–Wilk test, and homogeneity of variance was tested using Levene’s test. A two-way analysis of variance (ANOVA) was performed to evaluate the effects of treatment, sampling period, and their interaction. *Post hoc* comparisons were conducted using Duncan’s multiple range test at a 5% significance level (P ≤ 0.05) with the ‘agricolae’ package. All statistical values are presented as mean ± standard error, and means sharing the same letter within each column are not significantly different according to Duncan’s test. All figures were generated using the ‘ggplot2’ package in R.

## 3 Results

### 3.1 Evaluating the efficiency of the microbial consortium and compost in managing disease severity

**Figure 2:**
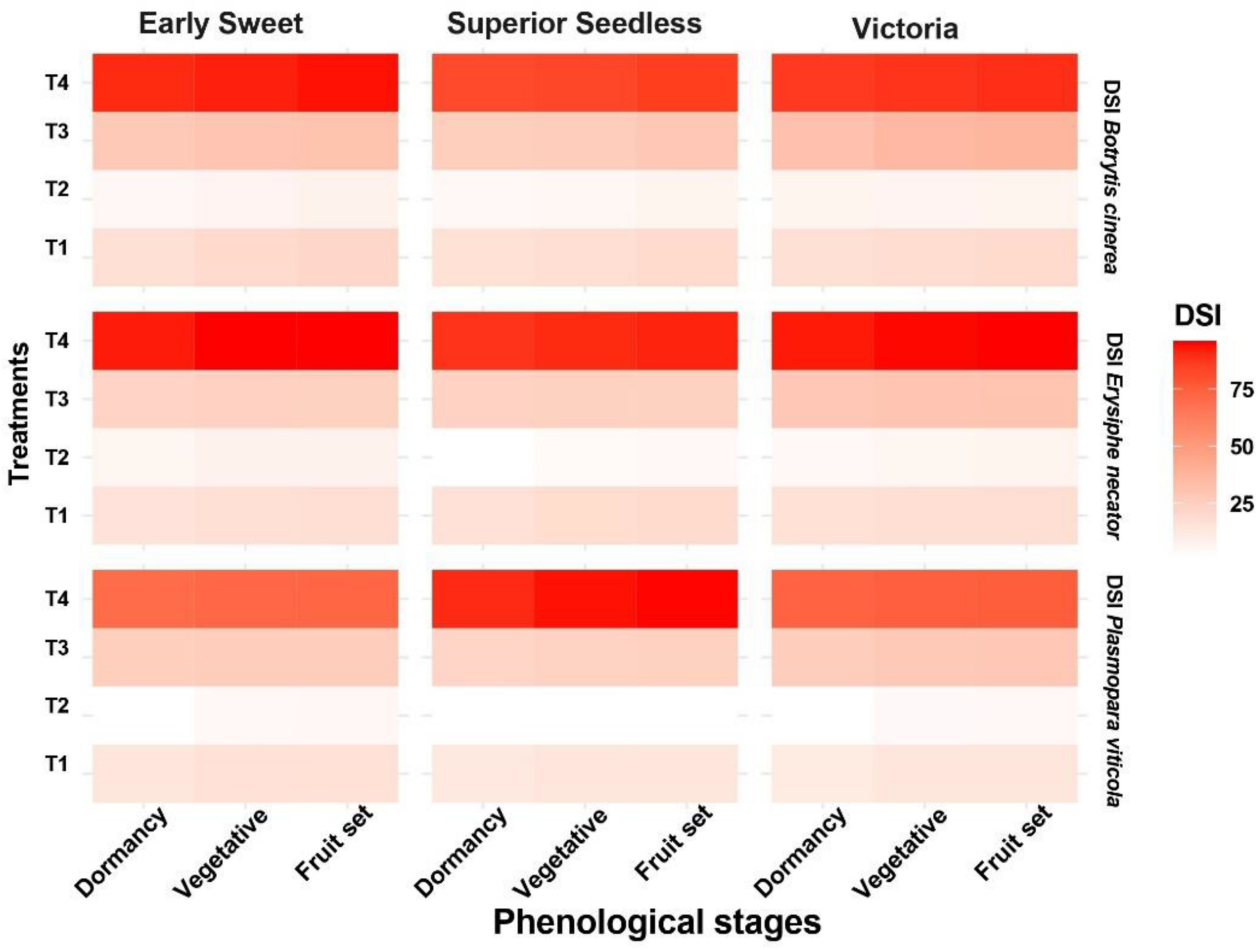
Heatmap of treatment effects on disease severity index (DSI) rates of *Botrytis cinerea*, *Erysiphe necator*, and *Plasmopara viticola* across the different phenological stages on Victoria, Superior Seedless, and Early Sweet cultivars (T1: compost; T2: microbial consortium + compost; T3: microbial consortium; T4: negative control).

**Figure 3:**
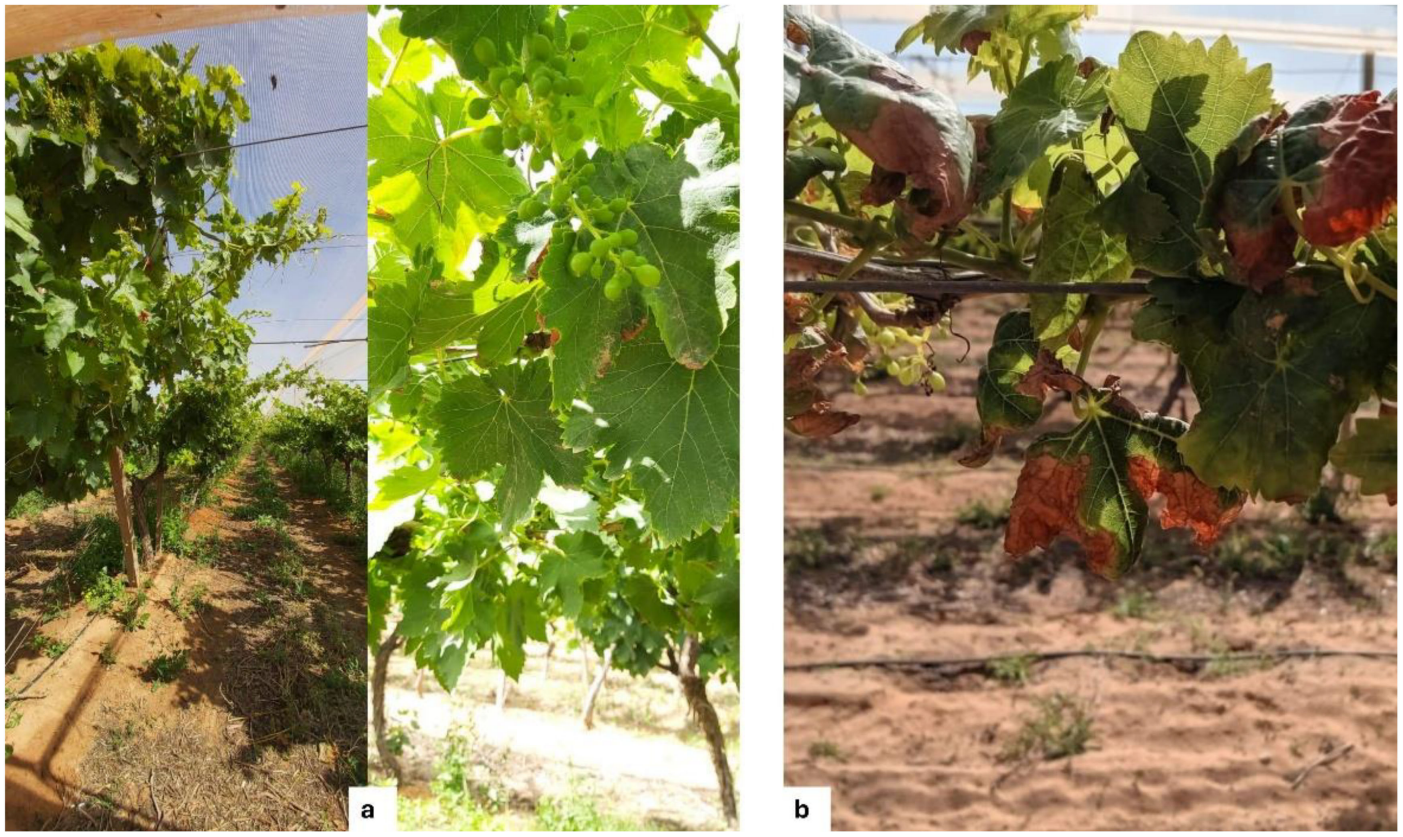
Visual comparison of grapevine leaves following treatment with compost and the microbial consortium. Leaves from a treated plant **(a)** show healthy, green foliage, while leaves from an untreated control plant **(b)** exhibit disease symptoms.

All grapevine cultivars in this study showed varying levels of susceptibility to fungal diseases. Based on the negative controls (T4), the three cultivars exhibited similar susceptibility to *B. cinerea* and *E. necator* across all phenological stages. However, for *P. viticola*, Superior Seedless consistently showed the highest disease severity and was therefore considered the most susceptible cultivar (**Figure 2**). Among the applied treatments, during dormancy, vegetative growth, and fruit set, T2 the combination of the microbial consortium and compost reduced disease severity more effectively than other treatments. Severity of *B. cinerea* decreased from 82%–92% to 4.97%–7.71%, *E. necator* from 89%–95% to 2.34%–8.03%, and *P. viticola* from 70%–95% to 2.84%–5.66%, representing reductions of up to 97%. Statistical analysis confirmed that all treatments significantly reduced disease severity across cultivars and phenological stages compared with the control (T4) (P < 0.01; **Supplementary Table S1**). Although individual applications of compost or the microbial consortium also reduced disease severity, their effects were less pronounced than those of the combined treatment (**Supplementary Table S1**, **Figure 3**).

ANOVA analysis means of a column followed by the same letter are not significantly different according to the Duncan’ test (P<0.05). Means ± standard error. (T1: compost. T2: microbial consortium + compost. T3: microbial consortium; T4: negative control).

### 3.2 Chlorophyll content

**Figure 4:**
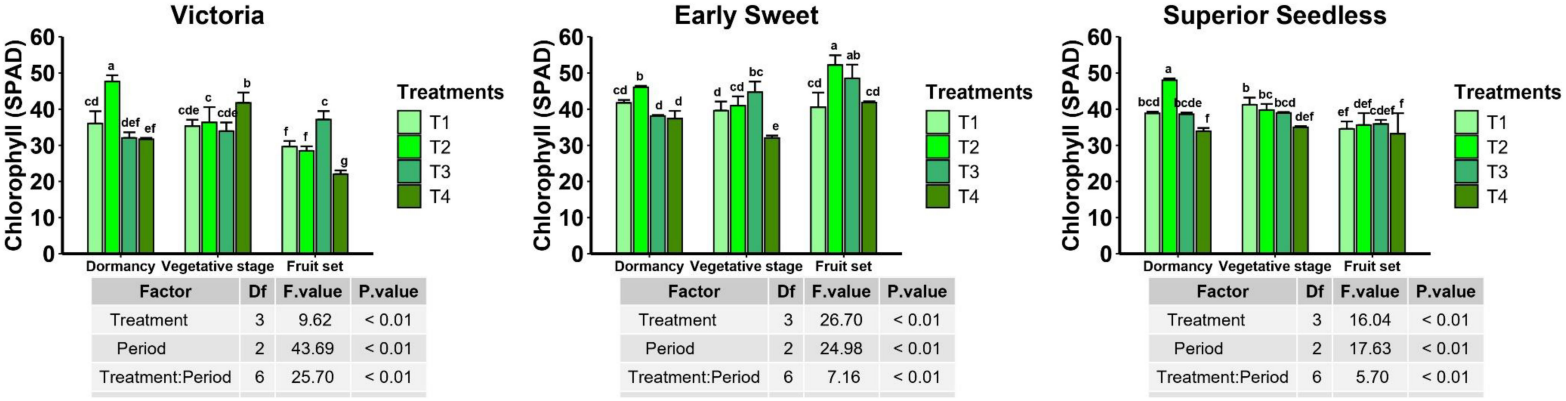
Grapevine leaf chlorophyll content following microbial consortium and compost treatments during dormancy, vegetative growth, and fruit set in the Victoria, Early Sweet, and Superior Seedless cultivars. Two-way ANOVA with Treatment × Period interaction, followed by Duncan’s multiple range test (P ≤ 0.05), where treatments sharing the same letters are not significantly different. Data are presented as mean ± SE (n = four leaves x six plants). (T1: compost; T2: compost + microbial consortium; T3: microbial consortium; T4: negative control)3.3 Enzyme activities and stress-related parameters.

Chlorophyll content (SPAD values) varied significantly in response to treatment, cultivar, and phenological stage, with a strong treatment × stage interaction (P < 0.01) (**Figure 4**). Treatments involving microbial applications (T2 and T3) were key contributors to this interaction, significantly influencing SPAD values depending on the cultivar and phenological stage. In Early Sweet, T2 (compost + microbial consortium) produced the highest chlorophyll content at fruit set (52.2), followed by T3 (48.5), highlighting its efficacy during this critical stage, while the control (T4) recorded the lowest values. During dormancy, T2 also exhibited the highest SPAD value (46.07), whereas the value under T3 (38.1) did not differ significantly from the control (T4, 37.4). For Superior Seedless, the strongest treatment effect was observed during dormancy, where T2 yielded the highest SPAD content (48.0), while a consistent but non-significant increase in SPAD values was observed under T2 and T3 compared to the control during the vegetative and fruit set stages. In Victoria, a slightly different pattern was observed. T2 generated the highest chlorophyll content during dormancy (47.63), while the control (T4) unexpectedly showed elevated chlorophyll levels during the vegetative stage (41.73). By fruit set, T3 produced the highest chlorophyll content (37.13), significantly exceeding the control (27.28). Overall, these results demonstrate that the impact of biologically enriched soil amendments is cultivar- and stage-dependent. However, treatments involving microbial consortia particularly the combined compost–microbial treatment (T2) consistently enhanced chlorophyll levels at key developmental stages, supporting their potential as a sustainable cultivation strategy.

#### 3.3.1 Ascorbate peroxidase activity

**Figure 5:**
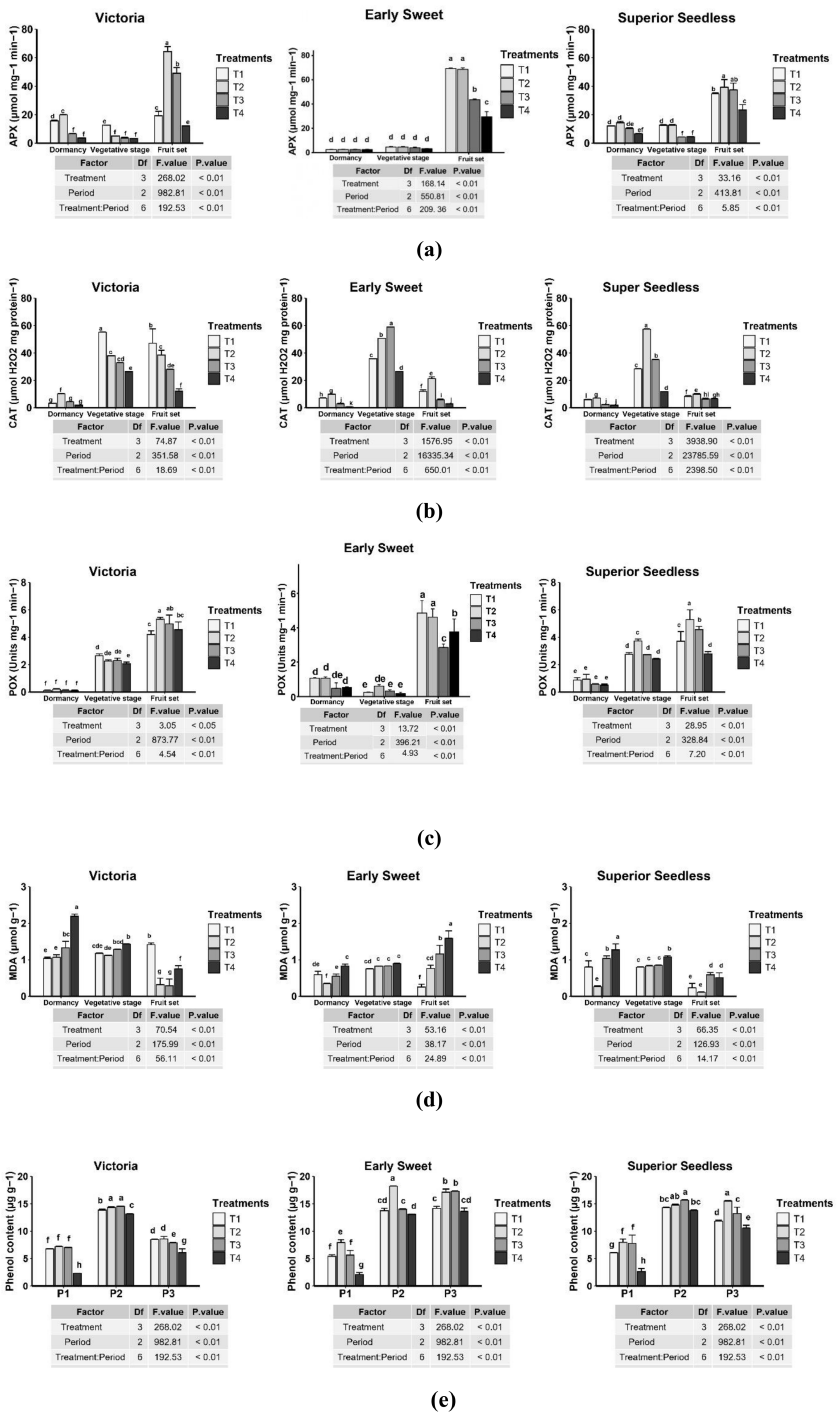
Impact of a microbial consortium of *Trichoderma longibrachiatum*, *Pseudomonas yamanorum*, and compost on plant defense mechanisms and stress markers in grapevine cultivars: APX, ascorbate peroxidase **(a)**; CAT, catalase **(b)**; POX, peroxidase **(c);** MDA, malondialdehyde **(d)**; phenolic content **(e)**. Different letters above the error bars indicate significant differences among treatments (P < 0.05) according to Duncan’s test; treatments sharing the same letter are not significantly different. (T1: compost; T2: microbial consortium + compost; T3: microbial consortium; T4: negative control).

APX activity showed significant differences among cultivars, treatments, and phenological stages, with strong treatment × stage interactions (P < 0.01) (**Figure 5a**). In the Victoria cultivar, APX activity during dormancy (P1) was highest under T2 (19.89 µmol mg⁻¹ min⁻¹), while T3 and T4 showed the lowest values. At the vegetative stage (P2), T1 (12.65) maintained higher activity compared with T3 and T4. At fruit set (P3), APX activity increased markedly under T2 (64.32), followed by T3 (49.08), whereas T1 and T4 remained significantly lower.

In Early Sweet, no significant differences were observed among treatments at P1, where values ranged from 2.5 to 2.58. At P2, values remained similarly low (3.2–4.6), indicating a weak antioxidant response during early stages. However, at fruit set (P3), APX activity increased substantially under T1 (69.27), T2 (68.60), and T3 (43.57), while the control (T4) showed the lowest value (29.48).

In Superior Seedless, moderate APX levels were recorded at P1, with T2 (14.33) and T1 (12.11) higher than the control (6.67). At P2, compost (T1) and the combined treatment (T2) maintained higher activities (~12.6), whereas T3 and T4 were significantly lower and similar to one another. At P3, the combined treatment (T2) produced the highest APX activity (39.25), followed by T3 (37.43), which exceeded T1 (34.67) but without significant difference, while T4 (23.35) remained the lowest. APX activity was strongly induced at fruit set (P3), particularly under compost combined with microbial consortium (T2) and microbial consortium alone (T3). These results suggest that microbial inoculation with or without compost enhanced antioxidant defense and improved protection against oxidative stress during late development.

#### 3.3.2 Catalase activity

Catalase activity considered as a crucial marker for assessing the efficacy of preventive treatment against plant disease and an important indicator of oxidative stress. The ANOVA analysis showed that CAT activity varied significantly among cultivars, treatments, and phenological stages, with strong interactions (p < 0.01) (**Figure 5b**). In the Victoria cultivar, compost (T1) consistently stimulated the highest CAT activity, particularly at the vegetative stage (55.20 µmol H_2_O_2_ mg protein⁻¹) and fruit set (47.07). The combined treatment (T2) also enhanced CAT activity (37.92 at P2; 38.49 at P3), whereas T3 and T4 remained lower, especially at P3 (28.08 and 12.08, respectively).

In Early Sweet, the strongest response occurred under T3 at the vegetative stage (58.94). T2 exhibited higher values at dormancy (9.88) and fruit set (21.34) compared with the control, which recorded 0.52 at P1, 26.5 at P2, and 2.88 at P3. Compost (T1) also promoted CAT activity at P2 (35.82) but was less effective than T2 and T3; however, T1 produced significantly higher activity than the control in all three stages. In Superior Seedless, the combined treatment (T2) exhibited the highest CAT activity across stages, with a peak at P2 (57.30). Compost (T1) and T3 showed moderate effects, while the control remained lowest across all stages. Across cultivars, CAT activity was highest during the vegetative stage (P2), indicating this phase as critical for antioxidant defense activation. Responses were cultivar-specific: compost (T1) was most effective in Victoria, the microbial consortium (T3) was most effective in Early Sweet, and the combined treatment (T2) was most effective in Superior Seedless. These findings highlight the complementary roles of compost and microbial inoculants in enhancing grapevine antioxidant defenses.

#### 3.3.3 Peroxidase activity

Across all three cultivars, peroxidase activity varied significantly with both treatment and phenological stage (P < 0.01) (**Figure 5c**). At P3, the highest POX activity was recorded under the combined compost–microbial consortium treatment (T2) in Victoria (5.32 units mg⁻¹ min⁻¹) and Superior Seedless (5.29 ± 0.71). In Early Sweet, compost alone (T1; 4.85) resulted in the highest POX activity, though it did not differ significantly from the combined treatment (T2; 4.61). In contrast, the negative control (T4) generally showed lower POX levels; however, this pattern was not consistent across all cultivars and stages, and in some cases POX values under T4 were equal to or higher than those under certain treatments. These findings suggest that the combined compost–microbial consortium treatment (T2) was most effective in enhancing grapevine defense particularly in Victoria and Superior Seedless while Early Sweet showed a stronger response to compost alone.

#### 3.3.4 Malondialdehyde content

The ANOVA analysis revealed a strong interaction that varied significantly among treatments, cultivars, and phenological stages (P < 0.01) (**Figure 5d**). The pattern of MDA accumulation was cultivar dependent. Results showed that MDA levels decreased in most cases from dormancy (P1) to fruit set (P3) in the Victoria and Superior Seedless cultivars most notably under the T2 and T3 treatments. However, the opposite trend was observed in Early Sweet, where MDA content increased across phenological stages. For the Victoria cultivar, the negative control (T4) recorded the highest MDA values at both P1 (2.19 µmol g-1) and P2 (1.43), while the lowest values were recorded at fruit set (P3) under T2 (0.32) and T3 (0.29). In Early Sweet, the compost treatment (T1) produced the lowest MDA content at fruit set (0.25), whereas the negative control showed the highest accumulation at the same phenological stage, accounting for 1.59. Similarly, in the Superior Seedless cultivar, the combined treatment T2 considerably reduced MDA levels at P1 (0.27) and at fruit set (P3, 0.11), in contrast to the negative control, which showed higher values across stages, particularly at dormancy (1.28). Overall, during the fruit set stage, the combined compost–microbial consortium treatment (T2) and the microbial consortium alone (T3) were the most effective in lowering MDA levels. These findings suggest that, compared with the untreated control (T4), biological amendments may exert a protective effect by reducing oxidative stress and lipid peroxidation in grapevine leaves.

#### 3.3.5 Phenolic content

Significant changes in phenolic content in grapevine leaves were recorded across cultivars and phenological stages in response to the different treatments (p < 0.01) (**Figure 5e**). In Victoria, the negative control (T4) consistently recorded the lowest phenolic levels at all phenological stages. The highest accumulation occurred at the vegetative stage (P2), where the combined compost–microbial consortium treatment (T2) accounted for 14.34 µg g-1, and the microbial consortium alone (T3) recorded 14.55. In Early Sweet, T2 induced the highest phenolic content at both P2 (18.24) and P3 (17.12), while T3 maintained high levels at fruit set (17.27). In Superior Seedless, T2 (15.51) and T3 (13.21) produced the greatest phenolic accumulation at fruit set. The negative control showed the lowest phenolic levels at P1 and P3, although at P2 its values did not differ significantly from those of T1 and T2 (P < 0.01). Overall, compost and microbial consortium treatments enhanced phenolic accumulation in grapevine leaves, with the strongest effects observed at the vegetative stage across all cultivars and at fruit set in Early Sweet and Superior Seedless.

## 4 Soil physicochemical responses to compost and microbial consortium application

**Figure 6:**
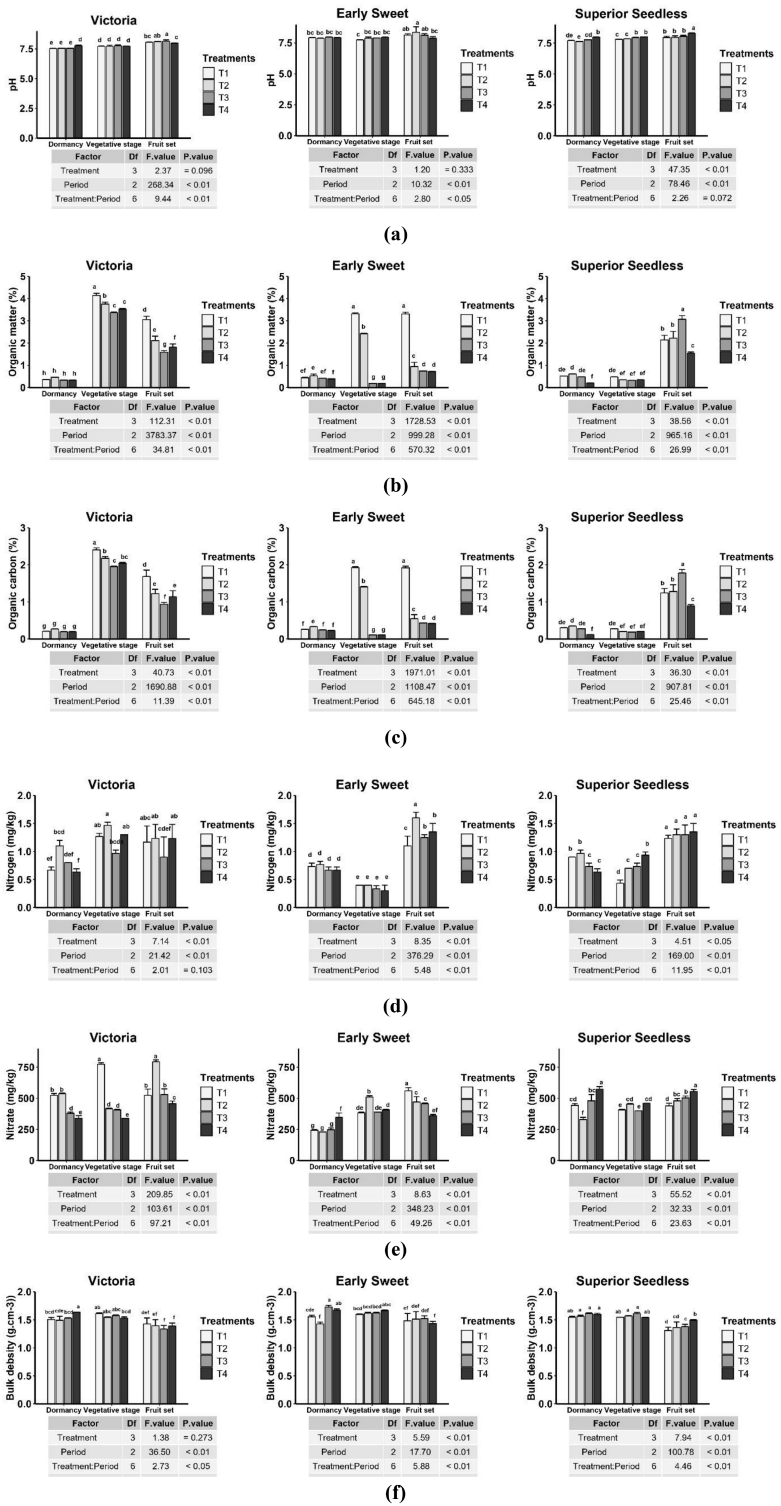
Changes in soil physicochemical properties of grapevine cultivars in response to treatments (T1: compost only; T2: compost + microbial consortium; T3: microbial consortium; T4: negative control). **(a)** pH; **(b)** organic matter; **(c)** organic carbon; **(d)** nitrogen content; **(e)** nitrate content; **(f)** bulk density. Different letters above error bars indicate significant differences between treatments (P < 0.05) according to Duncan’s test; treatments sharing the same letters are not significantly different.

In most cases, the combined application of compost and a microbial consortium (T2) produced the most pronounced improvements in soil physicochemical properties, with effects varying significantly by cultivar and phenological stage (P < 0.01 for major soil parameters; **Figure 6a**). Soil alkalinity generally increased from dormancy (P1) to fruit set (P3), with considerable differences observed at fruit set.

For Victoria, the combined treatments T2 (8.11) and T3 (8.15) resulted in a significantly more alkaline environment compared with the control T4 (7.97). In contrast, Superior Seedless soils were most alkaline under the control treatment (T4, 8.29) at P3. Early Sweet remained relatively consistent across treatments and stages, except at P3, where the combined compost–microbial treatment (T2) resulted in the highest pH (8.37). This suggests that amendment effects on soil pH may be influenced by grapevine cultivar.

The amendments by compost or combined treatment including compost and microbial consortium, impacted soil organic matter (OM) and organic carbon (OC) content, with effects that were distinctively cultivar-dependent (p < 0.01) (**Figures 6b, c**). In Victoria, all treatments markedly increased OM and OC levels at the vegetative stage (P2) compared with P1. At P2, compost (T1) generated the highest values (4.14% OM; 2.40% OC), significantly outperforming other treatments. In Early Sweet, compost (T1) produced the greatest OM and OC at P2 and P3 (3.32% and 1.92%, respectively). However, the combined treatment (T2) led to a notable decline by P3 (0.94% OM; 0.55% OC), suggesting a potential priming effect in which microbial activity accelerated decomposition of native soil organic matter. In the Superior Seedless cultivar, the microbial consortium (T3) demonstrated the most effective long-term impact, producing the highest P3 values (3.07% OM; 1.78% OC). This suggests that compost functions primarily as a direct organic amendment, whereas microbial inoculants may have different, sometimes opposing, effects depending on the cultivar either enhancing carbon sequestration through better plant growth or promoting decomposition. Soil nitrogen also increased in most cases under microbial consortium and compost treatments, though with cultivar-specific responses (P < 0.01) (**Figure 6d**). In Victoria, T2 produced the highest nitrogen level (1.47 mg/g at P2). In Early Sweet, T4 and T3 yielded the highest nitrogen by the fruit set stage (1.35 and 1.2, respectively). For Superior Seedless, all applied treatment performed similar trend by P3, which significantly enhanced nitrogen content above initial levels at P1. These findings revealed that the impact on nitrogen availability is highly dependent on the grapevine cultivar.

The availability of nitrate was also significantly varied on cultivar-specific response (**Figure 4e**). The Compost + consortium (T2) treatment, recorded the highest value for Victoria, T2 produced the highest nitrate content at P3 (793.33 mg/kg). Early Sweet showed the greatest nitrate content under T1 at P3 (560 mg/kg). By contrast, Superior Seedless showed no improvement under amendments, with the control (T4) retaining the highest nitrate levels (555 mg/kg). Regarding soil structure, ANOVA revealed a significant improvement in bulk density from dormancy (P1) to fruit set (P3) across all cultivars (P < 0.01) (**Figure 6f**). In Superior Seedless, treatment effects were highly significant (P < 0.01), with T1 showing the lowest final density (1.31 g/cm³) at P3.

For Victoria, bulk density decreased significantly from P1 to P3 (P < 0.01), indicating seasonal improvement in soil structure driven by natural factors such as root activity and weathering. However, amendment effects were not statistically significant (P = 0.273), as all treatments including the control showed similar density values at P3. A significant treatment × period interaction (P < 0.05) indicated that the pattern of seasonal change differed among treatments. However, at the fruit set stage (P3), *post-hoc* analysis revealed no statistically significant differences between any of the treatments (T1, T2, T3) and the control (T4).This implies that although amendments did not yield a particularly better result than the seasonal effect, they did have an impact on the dynamics of soil structure change over time (**Figure 6f**). For Early Sweet, at final stage of P3 all treatments increased compaction compared to the control, which yielded the least compact soil (1.43 g/cm³). Although densities under amendments were higher, the differences were not statistically significant, underscoring the importance of natural and cultivar-specific processes.

## 5 Discussion

This study examined the impact of various treatments on disease severity, chlorophyll content, stress-related biochemical parameters, and soil physicochemical properties in three economically important grapevine cultivars in Tunisia: Victoria, Early Sweet, and Superior Seedless. The findings highlighted significant cultivar-specific responses to the treatments and to pathogen pressure, demonstrating the strong interaction between cultivar, treatment, and disease progression.

The combined T2 treatment including compost, *P. yamanorum*, and *T. longibrachiatum* provided strong protection against *B. cinerea*, *E. necator*, and *P. viticola* across all three cultivars and phenological stages. Disease severity decreased from 82%–92% to 4.97%–7.71% for B. cinerea, from 89%–95% to 2.34%–8.03% for E. necator, and from 70%–95% to 2.84%–5.66% for P. viticola, representing reductions of up to 97% compared with untreated controls. These reductions exceeded those achieved by applying the microbial consortium or compost alone. Similar patterns have been reported in recent viticulture studies, where *Trichoderma* spp. reduced powdery and downy mildew severity by 60%–90% (Hajji-Hedfi et al., 2025b), and *Pseudomonas* spp. similarly pattern induced resistance against *P. viticola* (Bahmani et al., 2025). The disease reduction by T2 treatment across all cultivars and phenological grapevine stage in this study thus confirms that combining organic amendments with beneficial microbes can synergistically enhance biocontrol efficacy in vineyards (Valverde et al., 2025).

The physiology and growth of vines also reflected treatment effects. While disease control was evident, chlorophyll (SPAD) responses were cultivar specific. For instance, T2 resulted in the highest SPAD values at fruit set in Early Sweet and during dormancy in Superior Seedless, suggesting improved photosynthetic capacity under certain conditions. These observations are consistent with studies showing that microbial inoculants and compost can enhance crop vigor and chlorophyll content (Manjunath et al., 2023; Ouhaddou et al., 2024). Better nutrition and root function under the combination treatment are probably reflected in enhanced photosynthesis. In fact, when organic amendments are used to restore soil health, more chlorophyll (Lucchetta et al., 2023) is frequently observed (Lucchetta et al., 2023). The concept that these treatments increase vigor as part of disease resistance was supported by the T2 application, overall promotion of plant health, which extended beyond pathogen suppression. The compost and microbial consortium stimulated the grapevine’s defense system, including antioxidant enzymes (catalase, peroxidase, and ascorbate peroxidase) and total phenolic content were significantly elevated in treated plants. Beneficial bacteria can activate plant defense pathways, including phenylpropanoid metabolism and ROS-associated pathways, improving resistance capacity (Bahmani et al., 2025). For instance, *Pseudomonas fluorescens* can induce enzymes such as phenylalanine ammonia-lyase, polyphenol oxidase, and peroxidases, increasing antimicrobial phenolics and lignin precursors (Bahmani et al., 2025). Similarly, PGPR and fungal bio-stimulants frequently enhance APX, CAT, and SOD activity to mitigate oxidative stress (Hajji-Hedfi et al., 2025b; Li et al., 2025). In this study, increased CAT/POX/APX activity and elevated phenolic levels under T2 likely helped regulate ROS and reinforce cell walls, while lower MDA levels indicated reduced lipid peroxidation and cellular damage. These patterns are consistent with findings that *Pseudomonas chlororaphis* strains enhance grapevine defenses by upregulating defense genes and (Li et al., 2025). increasing SOD (superoxide dismutase), POD (peroxidase), and CAT activities, which in turn decreased downy mildew. The activation of antioxidant enzymes and secondary metabolites by beneficial microbes supports systemic resistance, which agrees with broader reviews (Hajji-Hedfi et al., 2025a; Hajji-Hedfi et al., 2025c).

Soil health was also enhanced under T2, and compost amendments, in particular when combined with BCAs, significantly improved soil organic matter, nutrient status and microbial activity. This was confirmed in our trial as increased soil organic carbon, available nitrogen and microbial biomass. These results correlate with prior research on vineyard composting. In fact (Lucchetta et al., 2023), demonstrated that applying compost for several years significantly increased soil organic matter (Lucchetta et al., 2023). and nutrient availability and increased the activities of microbial enzymes, particularly phosphorus-cycling enzymes, in vineyards. In the same context, pruning waste compost, particularly when fungus-inoculated, maintained increased soil enzyme activity and a more active microbial population in controlled trials compared to chemical fertilizer alone. In addition to enhancing organic matter, the combination treatment in our field tests also introduced *Trichoderma* and *Pseudomonas*), which can improve nutrient cycling and suppress soilborne pathogens (Hajji-Hedfi et al., 2023b). Cultivar-specific responses were notable across biochemical and soil parameters. Although all cultivars benefited from T2, the magnitude and pattern of responses differed. Superior Seedless showed the highest catalase and total phenolics, and the lowest MDA levels, under T2 treatment, indicating strong oxidative stress mitigation. Early Sweet displayed an early activation of defenses, whereas Victoria showed the largest increase in enzyme activity. These variations most likely result from biological varietal characteristics that influence how each cultivar responds to the addition of compost and biocontrol chemicals. In grapevines, cultivar diversity in induced resistance is well-established. Comprehending these varietal trends can help with targeted, variety-specific optimization may be beneficial.

The results have strong implications for sustainable viticulture and integrated disease management. Combining compost with beneficial microbes provides a non-chemical strategy that effectively controls major grape pathogens while improving soil fertility. Although this study was conducted in a single season in a Tunisian vineyard and soil and climate conditions may affect slightly the results: as (Bonanomi et al., 2025) emphasize, field results for organic amendments must be validated across multiple years and sites. Likewise (Hajji-Hedfi et al., 2025b), recommend multi-season trials in Vineyard to confirm long-term stability of (Bonanomi et al., 2025; Hajji-Hedfi et al., 2025b) disease control. Future work will be conducted on this approach in diverse vineyard sites and over several seasons to confirm durability. Nonetheless, the present findings indicate that compost combined with beneficial microbial consortia is a promising component of sustainable disease management in viticulture.

## 6 Conclusions

In this study, the combination of *T. longibrachiatum* and *P. yamanorum* with compost improved disease resistance, biochemical stress responses, and soil quality in grapevine cultivars. The treatment reduced disease severity, enhanced antioxidant enzyme activity, and increased phenolic levels. It also improved key soil parameters, contributing to healthier plants overall. These findings indicate that integrating compost with beneficial microbes may be used as a sustainable and effective strategy for managing disease and improving soil health in grapevine cultivation across different phenological stages.

## Data Availability

The datasets presented in this study can be found in online repositories. The names of the repository/repositories and accession number(s) can be found below: https://www.ncbi.nlm.nih.gov/genbank/, OP799680 https://www.ncbi.nlm.nih.gov/genbank/, PQ555427.

## Supplementary material

Supplementary Table 1 Effect of *Trichoderma longibrachiatum*, *Pseudomonas yamanorum* and compost application on disease severity of *Botrytis cinerea*, *Erysiphe necator*, and *Plasmopara viticola*.
