# Supplementary material for: Integrating beneficial microorganisms and soil amendment for grapevine health: toward eco-friendly seasonal fungal disease management and soil improvement
Source: Front Fungal Biol. 2025 Dec 10;6:1713132. doi: 10.3389/ffunb.2025.1713132 (PMC12728585; doi:10.3389/ffunb.2025.1713132)
Supplement: Supplementary Table 1 — Effect of Trichoderma longibrachiatum, Pseudomonas yamanorum and compost application on disease severity of Botrytis cinerea, Erysiphe necator, and Plasmopara viticola. [file Table1.docx]

**Table S1**. Effect of Trichoderma longibrachiatum, Pseudomonas yamanorum and compost application on disease severity of Botrytis cinerea, Erysiphe necator, and Plasmopara viticola

| Cultivars |  | Domancy | | | Vegetative stage | | | Fruit set | | |
| --- | --- | --- | --- | --- | --- | --- | --- | --- | --- | --- |
|  | **Treatments** | *Botrytis cinerea* | *Erysiphe necator* | *Plasmopara viticola* | *Botrytis cinerea* | *Erysiphe necator* | *Plasmopara viticola* | *Botrytis cinerea* | *Erysiphe necator* | *Plasmopara viticola* |
| Victoria | **T1** | 17.24±1.00c | 16.55±3.31c | 12.35±1.54c | 18.87±0.57c | 17.32±0.32c | 14.35±0.59c | 19.31±0.59c | 18±1c | 14.66±0.57c |
|  | **T2** | 7.71±0.56d | 5.35±2.31d | 3.36±2.08d | 7±1d | 6.66±0.57d | 4.65±0.51d | 7.66±0.57d | 7.66±0.57d | 4.66±0.57d |
|  | **T3** | 32.05±2.50b | 28.43±1.07b | 26.32±3.31b | 35.66±1.15b | 30.03±0.93b | 28.27±0.58b | 37.33±1.15b | 30.33±0.57b | 28.66±1.15b |
|  | **T4** | 86.68±8.01a | 93.47±2.17a | 73.48±5.28a | 88.63±0.55a | 95.19±1.30a | 74.80±1.02a | 89.66±0.57a | 95.66±1.15a | 75.66±1.15a |
|  | **P-Value** | **<0.01** | **<0.01** | **<0.01** | **<0.01** | **<0.01** | **<0.01** | **<0.01** | **<0.01** | **<0.01** |
| Early Sweet | **T1** | 16.82±1.41c | 15.36±2.88c | 14.67±2.07c | 20.33±1.52c | 17.07±0.40c | 16.47±0.53c | 21±1.73c | 18±1c | 16.66±0.57c |
|  | **T2** | 5.69±2.09d | 6.94±1.67d | 3.31±0.60d | 7±1d | 8.03±0.83d | 5.31±0.66d | 8.33±1.54d | 8±2d | 5.66±0.57d |
|  | **T3** | 28.05±0.89b | 22.72±1.58b | 25.19±3.20b | 29.66±0.57b | 24.18±1.15b | 26.29±1.07b | 31±1.73b | 24.66±0.57b | 26.33±1.52b |
|  | **T4** | 90.92±7.16a | 93.33±5.12a | 70.17±10.20a | 92.66±1.15a | 95.58±0.55a | 72.35±1.30a | 94.33±0.57a | 95.66±0.57a | 72.66±1.52a |
|  | **P-Value** | **<0.01** | **<0.01** | **<0.01** | **<0.01** | **<0.01** | **<0.01** | **<0.01** | **<0.01** | **<0.01** |
| Superior Seedless | **T1** | 16.69±1.55c | 16.02±3.02c | 13.38±3.21c | 17.33±0.57c | 19.12±0.38c | 14.34±0.36c | 19.33±1.15c | 19.33±0.57c | 14.66±0.57c |
|  | **T2** | 4.97±0.95d | 2.34±1.52d | 2.84±0.74d | 6±1d | 4.47±0.82d | 3.17±0.30d | 7.33±0.57d | 4.66±0.57d | 3.33±0.57d |
|  | **T3** | 25.74±2.01b | 23.69±1.50b | 21.86±2.23b | 26.66±0.57b | 24.45±0.56b | 23.43±0.47b | 28.66±0.57b | 24.66±0.57b | 24.66±0.57b |
|  | **T4** | 82.55±5.16a | 89.008±3.07a | 90.73±4.47a | 84±1a | 90.88±0.07a | 94.31±0.54a | 86±1.73a | 92±1.73a | 95.33±0.57a |
|  | **P-Value** | **<0.01** | **<0.01** | **<0.01** | **<0.01** | **<0.01** | **<0.01** | **<0.01** | **<0.01** | **<0.01** |

ANOVA analysis means of a column followed by the same letter are not significantly different according to the Duncan' test (P<0.05). Means ± standard error. (T1: compost. T2: microbial consortium + compost. T3: microbial consortium ; T4 : negative control).
